# Carbon-Based Polymer Nanocomposites for High-Performance Applications

**DOI:** 10.3390/polym12040872

**Published:** 2020-04-10

**Authors:** Ana Maria Díez-Pascual

**Affiliations:** Department of Analytical Chemistry, Physical Chemistry and Chemical Engineering, Faculty of Sciences, Alcalá University, 28871 Madrid, Spain; am.diez@uah.es; Tel.: +34-918-856-430

Carbon-based nanomaterials such as carbon nanotubes, graphene and its derivatives, nanodiamond, fullerenes, and other nanosized carbon allotropes have recently attracted a lot of attention among the scientific community due to their enormous potential for a wide number of applications arising from their large specific surface area, high electrical and thermal conductivity, and good mechanical properties [1,2]. The combination of carbon nanomaterials with polymeric matrices (i.e., thermoplastics, epoxies, conducting polymers, biopolymers, etc.) leads to new nanocomposites with improved structural and functional properties due to synergistic effects [3], with applications in a variety of fields, such as in electronics, energy storage, automobiles, aerospace engineering, biomedicine, and so forth. 

In particular, the properties of carbon-based polymer nanocomposites can be easily tuned by carefully controlling the carbon nanomaterial synthesis route and additionally the versatile synergistic interactions amongst the nanomaterials and polymers. In this regard, non-covalent and covalent approaches have been used to modify the surface of carbon nanomaterials with the aim of improving their dispersion and interfacial interactions [4,5]. The non-covalent strategies are based on the intermolecular interaction on the nanomaterial surface via physical adsorption and/or wrapping [6], though the nanomaterial–polymer interfacial interaction is typically weak, and this limits the effective stress transfer. These comprise solution mixing, melt-blending, and in situ polymerization. The solution method requires the dispersion of both the carbon nanomaterial and the polymeric matrix in a suitable solvent [7]. The melt-blending process involves the blending of the carbon nanomaterial into a molten polymer matrix under intense shearing [8]. Nanocomposites can also be prepared via in situ polymerization [9], in which the carbon nanomaterial is initially swollen by the monomer, and upon addition of the initiator, the polymerization begins by light irradiation or heat. The covalent method relies on the formation of a chemical bond between the polymer and the nanomaterial [10], leading to a strong interfacial interaction, though can disrupt the conjugated п system of the nanomaterial, hence modifying the properties. Thus, novel surface modifications of carbon nanomaterials are required in order to develop nanocomposites with improved properties compared with conventional composites. This Special Issue, with a collection of 14 original contributions and one review, provides selected examples of the most recent advances in the preparation and characterization of polymer nanocomposites incorporating carbon nanotubes and graphene or its derivatives for a variety of applications.

Graphene oxide (GO), the oxidized form of graphene, has attracted a lot of interest as nanoscale material [5]. It is solution processable, amphiphilic, and biocompatible, hence it can interact with biological cells and tissues [7]. This nanomaterial presents a large number of oxygenated groups, mainly epoxide, hydroxyl, and carbonyl on the basal plane and carboxyl acids on the edges, and can produce stable dispersions in water [11]. Further, it possesses outstanding strength and exceptional optical and electronic properties joint with high stability, flexibility, and optical transparency [12,13]. Nonetheless, it is insoluble in non-polar and polar aprotic solvents, which limits some applications. In this regard, different functionalization approaches have been carried out, like the reaction with organic hexamethylene diisocyanate (HDI) [14]. The resulting HDI-GO was mixed with conductive polymers, including poly(3,4-ethylenedioxythiophene):poly(styrenesulfonate) (PEDOT:PSS) [15], polyaniline (PANI) [16], and polypyrrole-3-carboxylic acid (PPy-COOH) [17], in different weight percentages via a simple solution casting method, and the nanocomposites were analyzed by different techniques to obtain information on the effect of the HDI-GO level of functionalization and percentage on the nanocomposite properties. The best properties were obtained for nanocomposites with HDI-GO contents in the range of 2–5 wt %. These nanocomposites are very suitable to be used in energy storage devices, fuel cells, batteries, supercapacitors, solar cells, touch panels, and so forth.

Other carbon-based nanomaterials, such as carbon nanotubes (CNTs), carbon dots (C dots), and carbon aerogels (CAGs) have also been mixed with different polymers for energy storage applications [18,19]. In particular, CNT-PANI and CNT-PPy have been used as cathodes into Li-ion batteries, leading highly efficient discharge ranges [20]. CNTs are also used as anodes for this type of batteries, though they exhibit very high capacity loss and low initial columbic efficiency. To solve this issue, CNTs can be prelithiated via cyclic voltammetry with a polymer coating such as p-sulfonated poly(allyl phenyl ether) (SPAPE) [21], which eliminates the initial irreversible capacity of the CNT electrodes. This is a simple, fast and inexpensive method to attain high performance flexible anodes for microbatteries.

Numerous approaches for the production of nanocomposites based on CNTs and conductive polymers have been developed [22,23]. A thoughtful issue in the development of these nanocomposites is the CNT tendency to aggregation. To prevent nanotube agglomeration, in situ oxidative polymerization in the presence of the CNTs can be carried out. The use of ultrasound guarantees a homogenous CNT dispersion in the reaction medium and inhibits coagulation during polymerization. In particular, works focused on the oxidative polymerization of aniline in the presence of CNTs have demonstrated that PANI forms a uniform coating on the CNT surface that results in strong п–п interactions between the quinoid units of PANI and the aromatic rings of the CNTs [24]. This improves the electron transport in the nanocomposite and consequently the electrical performance. Following a similar approach, hybrid nanocomposites were prepared by in situ oxidative polymerization of a conjugated polyacid, diphenylamine-2-carboxylic acid (DPAC) in the presence of SWCNTs in both alkaline and acidic media [25]. The influence of the reaction pH and the SWCNT content on the structure, morphology, thermal stability, and electrical properties of nanocomposites was assessed. The nanocomposites displayed good electrical conductivity and thermal stability, and are suitable for application in supercapacitors, rechargeable batteries, sensors field-emission devices, and dye-sensitized solar cells.

On the other hand, a polyacrylamide-alginate hydrogel was mixed with different amounts of multiwalled carbon nanotubes (MWCNT) to develop a flexible and durable electrode. The electrical conductivity and the mechanical characteristics of the electrode (tensile strength and stretching modulus) gradually increased with increasing MWCNT concentration, with an optimal concentration at 2.79 wt %. Further, the thermal stability of the electrode improved upon raising the nanotube content. These nanocomposite films exhibited an interfacial double-layer capacitance at the highest nanotube content, corroborating their suitability for lightweight flexible energy storage applications [26].

Flexible pressure sensors have also attracted a lot of interest due to their potential in flexible robots, wearing electronic apparatus and electronic skins [27]. Amongst them, piezoresistive sensors are the most preferred due to their easiness of manufacture and simple collecting signal output [28]. A typical method to prepare piezoresistive sensors is to fabricate nanocomposites with an elastomer like polyurethane (PU) as matrix and conductive carbon nanomaterials like reduced graphene oxide (rGO) as a filler [29]. In this regard, an rGO/PU nanocomposite was fabricated by soaking an rGO aqueous solution with a PU foam and subsequently reduced with hydrazine vapors. This manufacturing process is cheap and highly reproducible. The developed sensor exhibited a simple structure, good sensitivity, durability, cycling stability, and fast response, hence it can be applied in piezoresistive devices, leading to outstanding sensing performance. Nonetheless, the properties of this type of nanocomposites are frequently limited by the heterogeneous graphene dispersion within the PU matrix. To solve this issue, an ionic liquid can be used as a dispersing agent [30]. The resulting nanocomposites exhibited a lower electrical percolation threshold, about 2 wt %, than the counterparts with pristine graphene, together with higher conductivity values. Further, the mechanical properties, namely Young’s modulus and tensile strength, also improved while the ductility was maintained. Further, electrostatic spraying technique has also been used to improve the dispersion of nanofillers like MWCNTs within a PU coating as matrix [31], and to enhance the antistatic and mechanical properties. Besides the MWCNT distribution, the surface hardness and wear resistance of the nanocomposites were significantly improved. The optimal wear resistance was attained for the nanocomposite with only 0.3 wt % MWCNT.

Thermoelectric generators comprising flexible and lightweight p- and n-type single-walled carbon nanotube (SWCNT)-based composites also display enormous potential for wearable electronic applications [32]. They are able to transform heat into electric energy and vice versa under a temperature gradient without moving parts. These generators are environmentally friendly, noiseless, and display a long lifetime, which makes them highly interesting for power generation in electronic devices. In recent work, the thermoelectric performance of SWCNT flexible films doped with polyethyleneimine (PEI) and Nafion were assessed. The nanocomposites were manufactured via simple solution casting followed by vacuum filtration [33]. The SWCNT/PEI nanocomposites were switched from p- to n-type upon addition of PEI contents higher than 13 wt %. Furthermore, interconnected SWCNTs networks were produced due to an outstanding SWCNT dispersion and film formation. A thermoelectric generator with three thermocouples of p- and n-type SWCNT/PEI nanocomposites yielded an open circuit voltage of 17 mV and a maximum output power of 224 nW at 50 K, which are very encouraging results for wearable autonomous devices.

High-performance hybrid microfibers comprising hyaluronic acid and MWCNTs have been prepared by a wet-spinning method [34]. The matrix acts as a biosurfactant and a crosslinker, thereby improving the MWCNT dispersion. Different factors influencing the nanocomposite properties including the MWCNT content, dispersion time and injection speed have been investigated. The hybrid with 1.4 wt % MWCNT showed outstanding mechanical properties, with a Young’s modulus as high as 9 GPa and tensile strength of 130 MPa, combined with superior flexibility and stability due to the excellent mechanical and electrical properties of MWCNTs. Thus, the matrix provides good mechanical support whilst the nanotubes offer a stable conductive path. The developed hybrid microfibers are highly suitable to be used in the fields of energy storage, micro devices, sensors, and intelligent materials. A wet spinning approach has also been used to develop polyacrylonitrile (PAN) grafted-amino-functionalized MWCNT nanocomposite fibers [35]. The nanocomposites were synthesized through in situ polymerization in aqueous solvent. The grafting degree of PAN onto the functionalized MWCNTs was determined as 73%. The nanocomposite fibers displayed a more compact structure and a more homogenous diameter distribution than the pristine fibers. Further, the addition of the grafted MWCNTs enhanced the level of crystallization, crystal size, tensile modulus, and strength of the PAN fibers. High-performance polymeric hybrids can also be developed by other approaches like a combination of in-mold decoration and microcellular injection molding [36], the method that minimizes the bubbles and improves the surface quality.

Self-healing polymeric materials hold the ability of being mended, which extends their self-life. They are based on the capacity of the linking moieties to split and reform after exposure to a stimulus, typically heat or light [37]. A lot of efforts have been devoted to the development of self-healing polymer nanocomposites incorporating nanofillers via in-situ polymerization to improve the nanofiller distribution, strength, modulus, and toughness. Recently, electrically induced self-healed nanocomposites incorporating CNTs have been developed as actuators and for electronic applications [38]. In this case, the healing process takes place by heat generation when an electrical current goes through a conductive matrix filled with a CNT network. The Diels–Alder reversible cycloaddition is one of the most common chemical reactions used for thermal healing. In this regard, electrically conductive self-healing nanocomposites consisting of MWCNTs dispersed into thermally reversible crosslinked polyketones have been developed [39]. The reversible nature relies on both covalent (Diels-Alder) and non-covalent (hydrogen bonding) interactions. The thermomechanical properties of the nanocomposites were easily tuned by modifying the MWCNT, diene-dienophile and hydroxyl moiety fractions. Nanocomposites with 5 wt % MWCNT exhibited up to 10^4^ S/m electrical conductivity, and reached temperatures in the range of 120–150 under 20–50 V. This novel method could be used to fabricate thermo-reversible, thermo-adhesive, electrically conductive, and self-repairing systems.

The combination of both inorganic nanoparticles and CNTs in a polymeric matrix is frequently used to obtain multifunctional materials with superior properties [40,41]. The nanoparticles such as TiO_2_, SiO_2_, ZnO, Ag, and nanoclays aid to enhance the physical and mechanical properties as well as flame retardant activity, thermal stability, and permeability, among others. Nanocomposites of polypropylene filled with both TiO_2_ nanoparticles and CNTs have been prepared via melt extrusion, with total nanofiller contents of 1, 5, and 10 wt % [42]. The thermal stability, Young modulus and electrical conductivity of the matrix increased while the level of crystallinity and thermo-oxidative degradation decreased with increasing nanofiller loading. The nanocomposites displayed improved flame retardancy, leading to a significant decrease of the peak heat release rate for nanofiller loadings of 5 and 10 wt %.

High-performance flexible supercapacitors based on a cellulose acetate membrane filled with both CNTs and Ag nanoparticles have been recently developed via a low-temperature, rapid and controlled direct writing method [43]. The Ag nanoparticles were uniformly dispersed within the CNTs, and both embedded within the porous structure of the cellulose membrane. The nanocomposite electrode showed outstanding electrochemical and mechanical electrochemical performance. The synergistic effect of both fillers led to enhanced conductivity, and also improved the specific volumetric capacitance. At an optimal concentration of 5 wt % of both nanofillers, excellent cycling stability was attained, with 75.92% capacitance retention. 
